# Thermosensory Roles of G Protein‐Coupled Receptors and Other Cellular Factors in Animals

**DOI:** 10.1002/bies.202400233

**Published:** 2024-12-26

**Authors:** Kohei Ohnishi, Takaaki Sokabe

**Affiliations:** ^1^ Physiology and Biophysics, Graduate School of Biomedical and Health Sciences (Medical) Hiroshima University Hiroshima Japan; ^2^ Section of Sensory Physiology, Center for Genetic Analysis of Behavior National Institute for Physiological Sciences Okazaki Aichi Japan; ^3^ Thermal Biology Group, Exploratory Research Center on Life and Living Systems National Institutes of Natural Sciences Okazaki Aichi Japan; ^4^ Graduate Institute for Advanced Studies, SOKENDAI Hayama Kanagawa Japan; ^5^ AMED‐PRIME Japan Agency for Medical Research and Development Tokyo Japan

## Abstract

In this review, we introduce the concept of “dual thermosensing mechanisms,” highlighting the functional collaboration between G protein‐coupled receptors (GPCRs) and transient receptor potential (TRP) channels that enable sophisticated cellular thermal responsiveness. GPCRs have been implicated in thermosensory processes, with recent findings identifying several candidates across species, including mammals, fruit flies, and nematodes. In many cases, these GPCRs work in conjunction with another class of thermosensors, TRP channels, offering insights into the complex mechanisms underlying thermosensory signaling. We examine how GPCRs function as thermosensors and how their signaling regulates cellular thermosensation, illustrating the complexity of thermosensory systems. Understanding these dual thermosensory mechanisms would advance our comprehension of cellular thermosensation and its regulatory pathways.

## Graphical abstract

Recent studies have revealed GPCR functions in temperature sensation. This article introduces dual thermosensing via GPCRs and TRP channels associated with the intracellular signaling components and cellular factors such as lipid interactions and pH changes, highlighting the complexity of the temperature sensing mechanism.

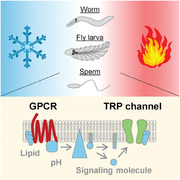

## 1 Introduction

Temperature significantly influences life events by modulating biological processes. Cell thermosensory mechanisms involve detecting temperature changes, transducing temperature information, and eliciting cellular or behavioral responses through the coordinated action of various cellular components. Temperature dynamics are mediated by multiple sensory molecules in the cell membrane, along with intracellular signaling components and the environmental factors, which are essential for highly sensitive thermosensation in cells. In higher organisms, many thermosensors have been identified in ion channels, including transient receptor potential (TRP) channels, whose thermal sensitivity can be modulated by G protein‐coupled receptors (GPCRs). Recent evidence suggests that GPCRs act as potential thermosensors with TRP channels, paving the way for a deeper understanding of thermosensation mechanisms. In this review, we introduce the properties of several known sensors in thermosensory pathways and examine the role of GPCRs in temperature signaling and thermosensation in mammals, Drosophila, and nematodes. We emphasize the concept of “dual thermosensory mechanisms,” highlighting the comprehensive roles of GPCRs and ion channels in precise thermosensory signaling. Additionally, we discuss the factors underlying the thermal sensitivity of GPCRs and the contributions of other cellular components or parameters (e.g., enzymes, lipids, and pH) to thermosensory signaling.

### 1.1 Temperature Sensitivity of Biological Molecules

Temperature affects various cellular functions, such as the central dogma, molecular composition, membrane fluidity, protein folding, and enzymatic activity [1]. Svante Arrhenius proposed the relationship between temperature and the rates of chemical reactions, providing an understanding of how temperature affects chemical reaction dynamics [2]. Q10 is a value that is commonly used to quantify the temperature dependency of biological processes; this value is defined as the ratio of the rates of a process at two temperatures that differ by 10°C [3]. For example, typical cellular processes have Q10 values ranging from 1 to 3, as dictated by the laws of chemistry. In contrast, achieving higher Q10 values requires special adaptations found in some proteins. In the case of ion channels, the rate of channel opening and closing shows Q10 values between 2.4 and 4, while maximum channel conductance shows Q10 values between 1.2 and 1.5 [4, 5]. Although “temperature activation” lacks a strict definition, it is clearly illustrated by thermosensitive TRP channels (thermo‐TRPs), which exhibit significantly higher Q10 values (approximately 10 or higher) than other biological molecules [6].

### 1.2 Thermosensitive Ion Channels

**FIGURE 1:**
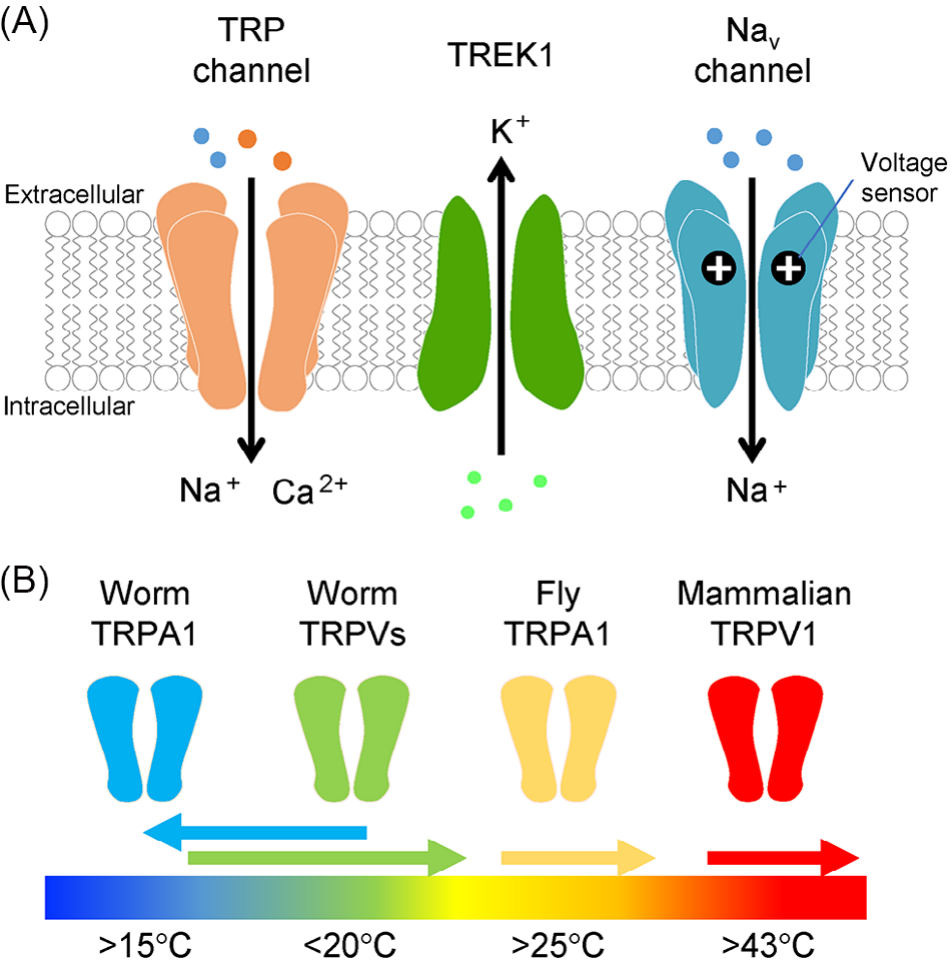
Thermosensitive ion channels expressed in nociceptive neurons. (A) Thermo‐TRPs are nonselective cation channels that function as thermosensors, responding to temperature changes as well as various compounds found in natural herbs and spices, along with other intrinsic and exogenous substances. K2P channels modulate background potassium currents to regulate neuronal activity. TREK‐1 responds to mechanical stimuli, lipids, pH, and temperature increase. Na_v_ channels are essential for neuronal excitation, with several exhibiting temperature‐sensitive voltage dependence and/or channel activity. Collectively, these channels play critical roles in sensory functions across various tissues, including pain perception and inflammatory responses. (B) Examples of thermo‐TRPs across species. TRP channels are widely conserved from nematodes to humans, although their responsiveness and thermal activation thresholds vary significantly among species. For instance, mammalian TRPV1 detects noxious heat above 43°C, while *C. elegans* TRPV channels OSM‐9 and OCR‐2 respond to increasing temperatures between 15°C and 35°C. TRPA1 acts as a heat sensor in many species, from insects to reptiles, whereas *C. elegans* TRPA1 functions as a cold sensor.

Nociceptive neurons contain various specialized receptors and ion channels, including TRP channels, two‐pore domain potassium (K2P) channels, and voltage‐gated sodium (Na_v_) channels (Figure 1A). In this section, we summarize the functions of these channels in thermosensation.

TRP channels and their associated genes are highly conserved across animal species, from nematodes to humans [7, 8, 9, 10]. TRP channels are classified into seven subfamilies—TRPC, TRPV, TRPM, TRPA, TRPN, TRPML, and TRPP—based on their amino acid sequences and structures [11, 12]. TRP channels are present in various cell types and tissues, playing significant roles in sensing surrounding environmental cues, including pressure, osmolarity, vision, taste, smell, sound, and temperature. Thermo‐TRPs are the most extensively studied thermosensors, activated by temperatures ranging from noxious cold to noxious heat, with many exhibiting unique temperature thresholds for activation [13, 14]. When the temperature exceeds or falls below a certain threshold, the open probability of thermo‐TRPs increases, generating activation currents caused by cation flux through the pore.

The first subtype of thermo‐TRPs, TRPV1, was identified in the subset of sensory neurons in rodents (Figure 1B) [15]. TRPV1 is a polymodal receptor that responds to various stimuli, such as capsaicin (an active ingredient in chili peppers), protons (low pH), and noxious temperatures above 43°C. The thermal sensitivity of thermo‐TRPs varies across species. For instance, in *Caenorhabditis elegans* (*C. elegans*), the TRPV homologs OSM‐9 and OCR‐2 function as heat sensors in sensory neurons, similar to mammalian TRPV1 [16].

On the other hand, TRPA1 exhibits diverse characteristics. While its chemical sensitivity to compounds, such as allyl isothiocyanate (a main ingredient in wasabi), is broadly conserved, its thermal sensitivity is highly species‐specific [17, 18, 19]. In mammals and nematodes, TRPA1 is considered to function as a cold sensor [18, 20, 21]; however, it is well established as a heat sensor in insects, amphibians, reptiles, and birds [22, 23, 24, 25, 26]. In *Drosophila*, TRPA1 is involved in various temperature‐related behaviors, such as thermal preference [27], with distinct functions of the isoforms adapted to specific needs [28].

K2P channels and Na_v_ channels are present in tissues involved in thermosensation. Within the K2P channel family, TWIK‐related potassium (TREK)‐1 and ‐2, and TWIK‐related arachidonic acid‐stimulated potassium (TRAAK) channels are temperature‐sensitive, with their activity rising 7–20 fold with a 10°C increase within the 10°C–40°C temperature range [29, 30, 31]. TREK‐1 is abundant in the central nervous system, playing a crucial role in sensing pain, mechanical force, and temperature changes [29, 32, 33]. Electrophysiological analysis showed that the outward‐rectifying potassium currents of TREK‐1 are significantly enhanced at temperatures above 30°C compared to those between 10°C and 20°C, and it was suggested that the C‐terminus may be involved in temperature‐dependent regulation of channel gating [29]. This C‐terminus mediates response to various stimuli, including chemicals, membrane stretching, arachidonic acid, and pH changes, indicating that multiple activating factors may interact with C‐terminus [34]. These factors may also affect each other to influence the activation of the receptor. For instance, it is suggested that temperature increase and membrane stretching synergistically enhance the activity of TREK‐1 through a shared mechanism in the C‐terminal domain to control channel gating. Furthermore, the activity of TREK‐1 is modulated by phosphorylation of its C‐terminus through GPCR signaling pathways [35, 36, 37]. For instance, serotonin inhibits TREK‐1 through the 5‐hydroxytryptamine 4 (5‐HT_4_) receptor (GPCR), Gα_s_, and cyclic AMP/protein kinase A (PKA), which enhances the depolarization‐induced activation of other channels, such as TRPV1, in the same neurons. This interaction facilitates the excitability of sensory neurons and amplifies pain sensations.

The Na_v_ channel family is not directly activated by temperature changes, but its gating properties are highly temperature‐sensitive. The human genome has nine Na_v_ α‐subunits (Na_v_1.1–1.9) and four auxiliary β‐subunits, which play a crucial role in neural firing as triggers for action potentials [38, 39, 40, 41]. Na_v_1.7, Na_v_1.8, and Na_v_1.9 are involved in pain signaling at peripheral nerve endings [42, 43], with their activities significantly influenced by temperature changes. Na_v_1.7 enhances neuronal excitability in response to small depolarizations, and several naturally occurring gain‐of‐function mutations in Na_v_1.7 lead to a range of pain symptoms that exhibit temperature‐sensitive phenotypes [44]. For example, inherited erythromelalgia is thought to result from hypersensitivity of the voltage‐dependent gating mechanism in these mutants to temperature, causing hyperexcitability of nociceptive neurons [45, 46, 47]. Conversely, the activation and inactivation kinetics of Na_v_1.8 and Na_v_1.9 are much slower than those of Na_v_1.7, leading to persistent excitation of neurons. Na_v_1.8 remains active at temperatures as low as 10°C, essential for detecting pain in the noxious cold range [48]. Na_v_1.9, however, accelerates its activation and inactivation rates, achieving approximately a 4‐fold increase in peak current upon a 10°C temperature increase, thereby contributing to noxious heat sensation [49].

### 1.3 GPCRs as Regulators of Ion Channels

GPCRs are the largest family of transmembrane receptors responsible for receiving extracellular signals and mediating cell responses. They detect various stimuli, such as neurotransmitters, hormones, peptides, inflammatory mediators, and exogenous chemical and physical signals, playing crucial roles in functions such as vision, smell, taste, inflammation, and many other biological processes. When ligands bind to GPCRs, they induce a conformational change that activates trimeric G proteins composed of Gα, Gβ, and Gγ subunits. Gα subunits are classified into four types based on their sequence characteristics, including Gα_s_, Gα_i/o_, Gα_q/11_, and Gα_12/13_, each of which triggers distinct signaling pathways [50, 51]. These subunits form various trimer combinations, creating diversity in cell signaling and providing specificity to GPCRs and their downstream pathways [52]. For instance, G protein signaling regulates ion channels by activating or inhibiting enzymes and facilitating calcium release from intracellular stores. Additionally, G protein signaling can amplify downstream signaling, as demonstrated in phototransduction in *Drosophila* photoreceptor cells [53].

The sensitivity of nociceptors is modified in response to repeated stimulation or tissue injury, a process that involves thermo‐TRPs and regulation by GPCRs [54, 55, 56]. GPCRs in the peripheral endings of nociceptive neurons receive various neuronal modulators, such as neuropeptides, lipids, and other endogenous factors from tissues like immune and epithelial cells. This interaction triggers multiple signaling pathways in pruritus, pain, and inflammation. GPCRs sensitize or activate TRP channels by regulating phospholipases and kinases through Gα or Gβγ subunits. Sensitization results in increased channel activity and a lowered thermal threshold, enabling thermo‐TRPs to activate at innocuous temperatures that would not normally trigger these channels. For instance, bradykinin, a neuropeptide produced in response to tissue injury or inflammation, binds to its corresponding GPCR, activating Gα_q_ and phospholipase C (PLC). PLC hydrolyzes phosphatidylinositol bisphosphate (PIP_2_) to produce the metabolite diacylglycerol (DAG) and inositol 1,4,5‐trisphosphate (IP_3_), which lead to the phosphorylation and sensitization of TRPV1 through the activation of protein kinase C (PKC) and the release of calcium from intracellular stores through the IP_3_ receptor [57, 58, 59]. Additionally, calcium influx through TRPV1 activates TRPA1, further contributing to inflammatory responses [60, 61].

### 1.4 Involvement of Opsin in Thermosensation

Opsins are light‐sensitive GPCRs widely conserved across animal species and are involved in image perception, light detection, and many other light‐dependent biological processes [62]. In mammals, rods and cones are responsible for vision under dim and bright light conditions, respectively, with opsins binding 11‐*cis* retinal as a ligand [63]. Light exposure converts 11‐*cis* retinal to all‐*trans* retinal, inducing a conformational change in opsin and activating a trimeric G protein. This activation stimulates cyclic GMP (cGMP) phosphodiesterase, closing the cGMP‐gated ion channels. While rods and cones were traditionally considered the sole retinal photoreceptors in mammalian eyes, light‐sensing capabilities have been found in other tissues, such as intrinsically photosensitive retinal ganglion cells (ipRGCs) and the sphincter muscles of the iris. These cells mediate photoentrainment of circadian rhythms and pupillary constriction, respectively [64, 65]. In both ipRGCs and sphincter cells, photopigment melanopsin is involved in light responses. Furthermore, melanopsin and related opsins are expressed in nonphotoreceptor cells, contributing to various nonimage‐forming light reactions. These functions are also found in other species, such as fish and mollusks with diverse downstream signaling pathways [66, 67].

**FIGURE 2:**
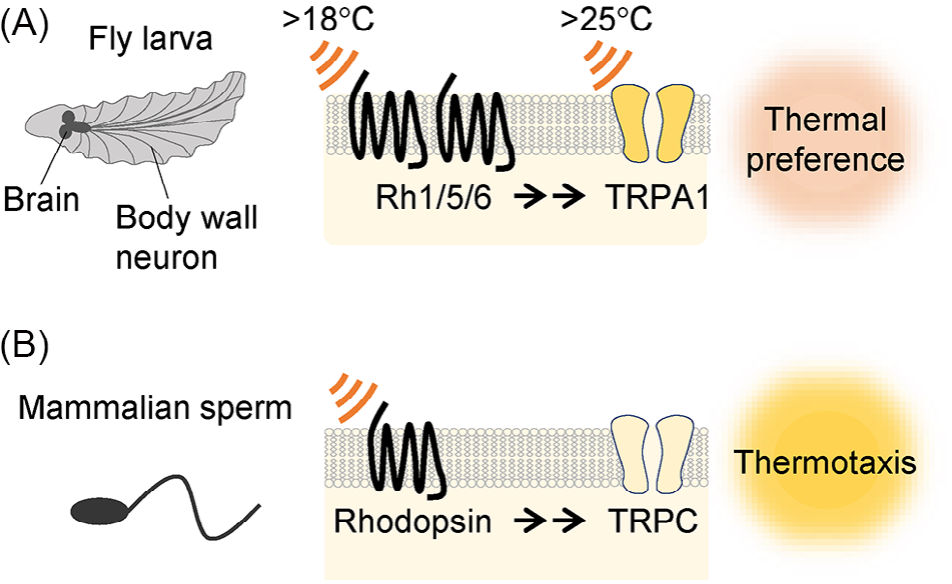
Temperature signaling through opsins and TRP channels in *Drosophila melanogaster* larvae and mammalian sperm. (A) Opsins and TRPA1 channels regulate the thermal preferences of *Drosophila* larvae. Multiple opsins may act as thermosensors, enabling discrimination between 18°C and higher temperatures up to 24°C in brain and body wall neurons. Temperatures above 25°C directly activate TRPA1. (B) Opsins and TRPC channels regulate the thermotaxis of mammalian sperm. Multiple opsins, expressed in distinct regions of sperm, can trigger cyclic nucleotide and PLC signaling cascades, which subsequently regulate downstream cyclic nucleotide‐gated channels and TRPC channels.

Recent studies have uncovered unexpected roles of opsins in thermosensation and temperature‐related behaviors in species such as fruit flies, honeybees, mosquitoes, and mammalian sperm. The involvement of opsin in thermosensory signaling was first identified in *Drosophila melanogaster* (fruit flies) [68]. At their larval stage, *Drosophila* exhibits a preference for 18°C over warmer temperatures (19°C–24°C), with Rhodopsin 1 (Rh1) playing a crucial role in this behavior (Figure 2A). Mutants lacking *rh1* lose the ability to differentiate between 18°C and temperatures up to 24°C. However, their aversive responses to colder (16°C or lower) and warmer (26°C or higher) temperatures remained intact. The thermosensory signaling pathway consists of Rh1, Gα_q_, a PLC (*norpA* in flies), and TRPA1, resembling the phototransduction cascade in mammalian ipRGCs [68, 69]. While temperatures above 25°C directly activate *Drosophila* TRPA1 [70], the channel is likely regulated downstream of PLC, similar to TRPC channels in ipRGCs, for discrimination around 18°C. Notably, although Rh1 functions as a light sensor in light‐sensitive organs in larvae, Rh1‐mediated thermotaxis is not affected by illumination.

Further analyses revealed that *Drosophila* larvae switch their thermal preference during development using multiple rhodopsins [71]. After hatching, larvae prefer around 24°C up to 72 h after egg laying (AEL), then switch their preference to 18°C between 96 and 120 h AEL before pupation. Besides Rh1, Rh5, and Rh6 also play key roles in this thermal preference switch. Rh5 and Rh6 are expressed in a subset of the central nervous system and peripheral sensory neurons, where Gα_q_, PLC, and TRPA1 are co‐expressed. Therefore, *Drosophila* larvae use the rhodopsin1/5/6–TRPA1 signaling pathway for temperature discrimination (Figure 2A). In adult flies, a similar pathway, including Rh6, PLC, and possibly a TRPV channel, helps detect cooler temperatures below 23°C, which suppress food intake [72].

Rhodopsin involvement in temperature‐related behavior has also been observed in honeybees. *Apis cerana japonica*, a honeybee species, exhibits unique defensive behavior against hornets by generating heat using their flight muscles, forming what is known as a “hot defensive bee ball” [73]. Worker bees form a ball‐like structure around a hornet and vibrate their flight muscles to increase the surrounding temperature to as high as 42°C, maintaining this heat for around 30 min to incapacitate the hornet. An RNA‐seq analysis revealed genes upregulated in the brain, flight muscles, and fat body during the bee ball formation [74]. Notably, rhodopsin and arrestin 2 were upregulated in all tissues. Additionally, PLC, arrestin 1, and carotenoid isomerooxygenase—molecules involved in rhodopsin signaling—were upregulated in the brain. Furthermore, a Hymenoptera‐specific TRP channel, HsTRPA, which exhibits heat sensitivity similar to *Drosophila* TRPA1 [23], was present in all tissues tested in the RNA‐seq analysis. Therefore, it is speculated that rhodopsin and its downstream molecules, including HsTRPA, are involved in temperature sensing in honeybees, and their upregulation is essential for the heat sensation and acclimatization necessary for maintaining bee ball behavior.

Recent studies have shown that mosquitoes possess infrared (IR) sensitivity to locate their hosts [75]. They detect body temperature‐derived IR radiation through two opsins and TRPA1 expressed in neurons at the tip of their antennae because the electrophysiological responses of neurons to IR radiation were diminished when either the opsins or TRPA1 were absent. The authors proposed that radiant energy induces local heating in the antennae, providing further evidence that rhodopsins may function as heat sensors.

Mammalian sperm exhibit thermotaxis, altering their swimming direction in response to temperature gradients and detecting temperature differences as low as 0.0006°C along their body length [76, 77]. Multiple opsins, including rhodopsin and melanopsin, are present in sperm and trigger multiple signal transduction pathways through cyclic nucleotides and PLC (Figure 2B). Mutations in rhodopsin resulted in defects in thermotactic behavior, highlighting the evolutionarily conserved role of rhodopsin and its downstream signaling cascades in temperature sensation.

### 1.5 Thermosensation Through GPCR and Ion Channels in *C. elegans*

**FIGURE 3:**
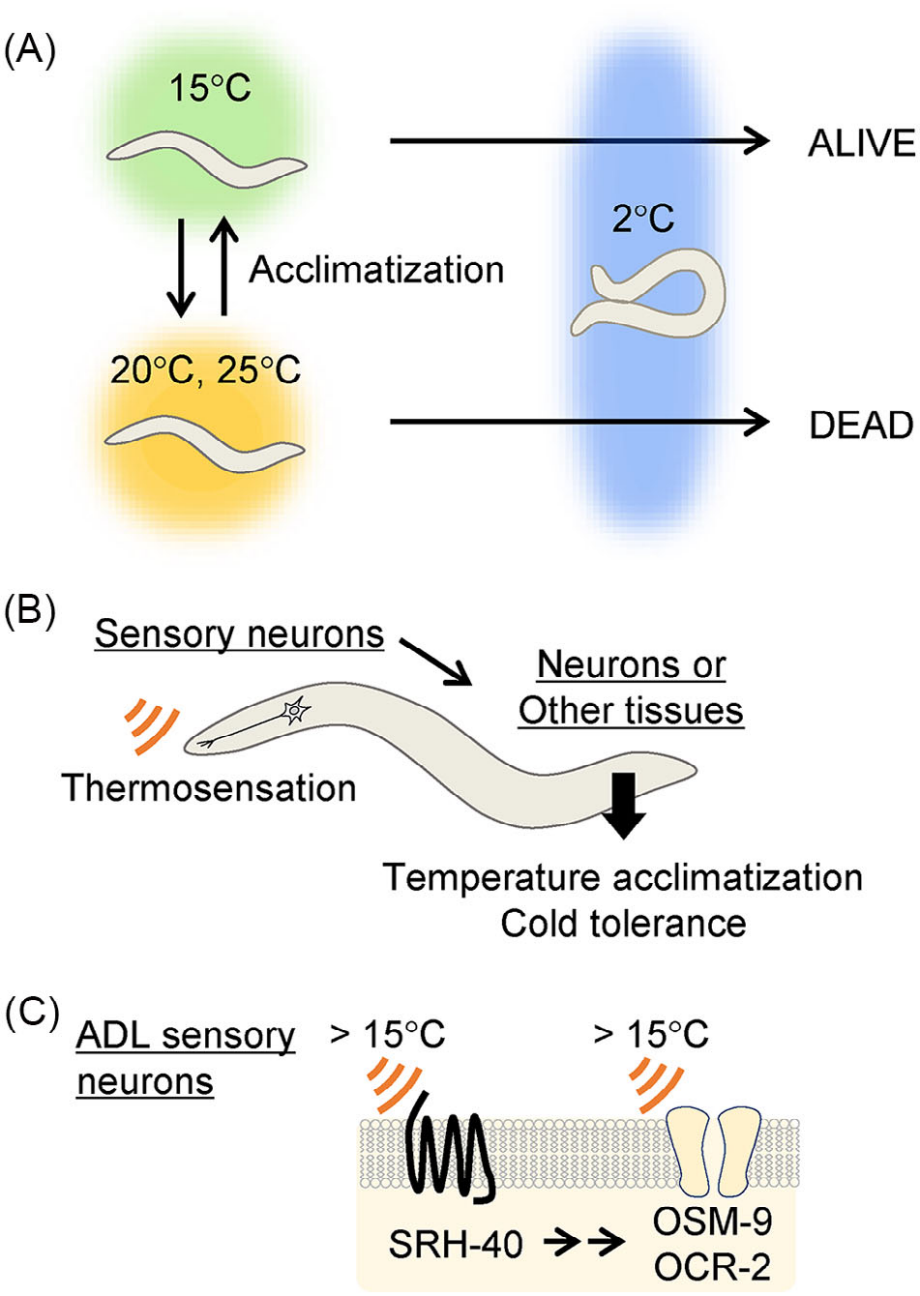
Mechanism of cold tolerance and temperature acclimatization in *C. elegans*. (A) *C. elegans* thrives at 15°C–25°C and can survive at 2°C when acclimated at 15°C; however, they perish when acclimated at 20°C or higher. This cold tolerance develops within 3–5 h of temperature acclimatization. (B) Cold tolerance following temperature acclimatization is regulated by thermosensation in multiple sensory neurons in the head, which transmit temperature signals to other neurons and tissues. (C) In ADL thermosensory neurons, the GPCR SRH‐40 functions as a thermosensor alongside the thermo‐TRPs OSM‐9 and OCR‐2.

The nematode *C. elegans* has been studied to investigate various thermosensory behaviors and their associated signaling pathways [78, 79, 80]. This organism exhibits cold tolerance, allowing it to survive at temperatures as low as 2°C, which depends on temperature acclimatization that occurs within 3−5 h at cooler cultivation temperatures (Figure 3A) [81, 82, 83]. These adaptations likely involve the regulation of several physiological processes coordinated by thermosensory neurons (Figure 3B). In the thermosensory ASJ neuron, G protein signaling—comprising guanylyl cyclase and cGMP‐gated channels—plays a crucial role in temperature signaling [81]. In the ADL chemosensory and thermosensory neuron, temperature acclimatization is mediated by the thermosensitive TRPV channels OSM‐9 and OCR‐2 [16, 82, 84], which may be regulated by PLC signaling. Therefore, GPCRs that function as thermosensors in these neurons have been anticipated for many years.

To identify GPCRs responsible for thermosensation in these neurons, we performed an RNAi screening assay in which we investigated the impacts of more than 1000 GPCRs on temperature acclimatization in *C. elegans*. We found that the loss of one GPCR (SRH‐40) that is expressed in ADL neurons led to a higher survival rate at 2°C than the control group after several hours of cultivation at 25°C, indicating that SRH‐40 is essential for acclimatization to warm temperatures [85]. The ectopic expression of SRH‐40 conferred thermal sensitivity in nonwarmth‐sensing neurons in *C. elegans* and *Drosophila* cultured cells, providing evidence that SRH‐40 may act as a thermosensor. These results indicate that two types of thermosensitive molecules—thermo‐TRPs and GPCR—collaborate within the same sensory neuron (Figure 3C). Calcium imaging analysis of the ADL neuron revealed that the *srh‐40* mutant, *osm‐9* and *ocr‐2* single mutants, and *srh‐40 ocr‐2* double mutant showed comparable and partial reductions in heat responses. This finding suggests that the SRH‐40 and TRPV channels may function within the same pathway. We also found that SRH‐40‐mediated temperature signaling requires Gα_q_, which activates the PLC pathway.

**FIGURE 4:**
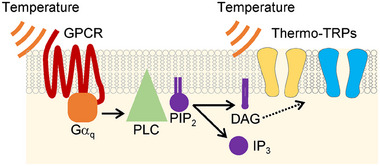
Dual thermosensory mechanism. This model illustrates a signaling pathway involving two thermosensors. When temperature stimulates GPCRs, PLC is activated by Gα_q_, producing DAG and inositol trisphosphate (IP_3_) from phosphatidylinositol bisphosphate (PIP_2_). DAG regulates the activity of thermo‐TRPs, which are also regulated by temperature changes, while IP_3_ stimulates IP_3_ receptors in the endoplasmic reticulum (not shown). A similar signaling pathway is present in inflammatory responses, where TRPV1 and TRPA1 are regulated by GPCR for inflammatory mediators. GPCRs, G protein‐coupled receptors.

**FIGURE 5:**
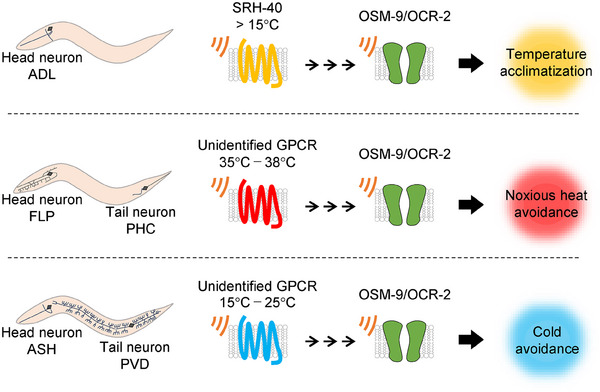
Examples of dual thermosensory signaling in *C. elegans*. In vitro electrophysiological analysis revealed that OSM‐9 and OCR‐2 function as thermosensors, responding to temperature increases between 15°C and 35°C. However, since OSM‐9 and OCR‐2 mediate thermosensation across various temperature ranges in different neurons, their activity likely depends on upstream signaling activated by different thermosensitive GPCRs. This may provide each sensory neuron with distinct thermosensing capabilities, allowing the organism to respond effectively to various temperature changes.

These findings, along with previous reports, lead us to propose the “dual thermosensory mechanism” involving two thermosensors whose functions may vary depending on the cellular environment (Figure 4). This hypothesis is supported by evidence that OSM‐9 and OCR‐2 play distinct thermosensory roles among different types of neurons, including ADL neurons (temperature acclimatization during a 15°C–25°C increase), PHC and FLP neurons (avoidance of noxious heat at approximately 35°C–38°C), and PVD and ASH neurons (avoidance of cold temperatures between 25°C and 15°C) (Figure 5) [16, 82, 86, 88]. Notably, ASH neurons respond to rapid cooling stimuli, and genetic analysis has shown that G proteins and OSM‐9 are involved in temperature signaling for cold avoidance [86]. While ASH neurons also respond to rapid and slow heating stimuli, these responses are weaker than those to rapid cooling stimuli [86, 89, 90].

The differences in the thermal sensitivity of OSM‐9 and OCR‐2 within and across neurons can be attributed to various modulations of these proteins through GPCR signaling. Temperature fluctuations activate either a single GPCR thermosensor or a combination, triggering distinct signaling pathways that differentially modulate OSM‐9 and OCR‐2. This aligns with observations in mammalian nociceptive signaling, where the phosphorylation of thermo‐TRPs by GPCR signaling induces channel sensitization and increases their localization in the membrane [54].

Beyond their role in dual thermosensory signaling, OSM‐9 and OCR‐2 are also activated in various sensory pathways. For instance, pheromonal stimulation of an as‐yet‐unidentified GPCR acts through the G_i_/G_o_‐family like protein GPA‐3 to activate OSM‐9 and OCR‐2, leading to the regulation of lipolysis in the intestine [91]. Lipolysis contributes to cold tolerance by reducing cold‐induced cellular damage in invertebrates, including *C. elegans* [92, 93], although its correlation with warmth temperature acclimatization remains unclear. Given that nonthermal stimuli can activate OSM‐9 and OCR‐2 through other GPCRs, the warmth temperature acclimatization likely relies on the SRH‐40‐OSM‐9 and OCR‐2 signaling pathways, highlighting the significance of these dual thermosensory mechanisms. Thus, the interplay between TRP channels and GPCR‐G protein signaling may allow for switches in their contributions to thermosensory and nonthermosensory signaling.

The dual thermosensing mechanisms involving GPCRs and TRP channels contribute to a complex and sophisticated thermosensation. It is intriguing to explore the unique properties of different GPCR signaling pathways and how they collectively affect temperature perception and related behaviors in animals. Furthermore, these dual thermosensory mechanisms may vary among species. In *Drosophila* thermotaxis, rhodopsin‐TRPA1 signaling detects temperatures between 18°C and 24°C, even though thermosensitive TRPA1 is not directly activated in this range (Figure 2A) [70, 71]. Conversely, both SRH‐40, OSM‐9, and OCR‐2, which are involved in temperature acclimatization in *C. elegans*, show comparable thermal sensitivities as follows: SRH‐40 is activated by heating from 15°C to 35°C in ADL neurons, and in vitro electrophysiological analysis shows that OSM‐9 and OCR‐2 are activated by heating within the same range (Figure 3C) [16, 85]. Thus, GPCRs and thermo‐TRPs may be activated in parallel in these contexts. Another mechanism involves GPCR signaling that is independent of temperature, which can modulate the thermal sensitivity of thermo‐TRPs, such as the sensitization of thermo‐TRPs as an inflammatory response described earlier. However, considering the examples of GPCRs functioning as thermosensors discussed so far, dual thermosensing by GPCR and downstream thermosensitive channels may enhance the precision and sensitivity of cellular thermal responses.

### 1.6 Temperature Sensitivity of GPCRs

While ion channel‐type thermosensors like thermo‐TRPs have been extensively studied, the thermal sensitivity of GPCRs remains unclear. Previous studies have examined the thermal stability of GPCRs, offering insights into how they may acquire thermal sensitivity and function as thermosensors.

Rhodopsin exhibits stable folding and was the first GPCR to be structurally analyzed, with bovine rhodopsin serving as the pioneering example [94, 95]. In spite of its thermal stability, rhodopsin can be stochastically activated, even in complete darkness, a phenomenon known as “dark noise.” To detect dim light and minimize background noise that may obscure light‐induced signaling, the heat‐dependent dark noise of rhodopsin is suppressed by multiple mechanisms. A covalent Schiff base linkage between 11‐*cis* retinal and opsin suppresses thermal isomerization of the 11‐*cis* retinal to the all‐*trans* form [96]. 11‐*cis* retinal acts as a covalently bound inverse agonist (deactivator) of opsin, and photon absorption converts it to all‐*trans* retinal, which serves as a covalently bound agonist (activator). This covalent binding between the ligand and opsin could be a special adaptation ensuring a much higher signal‐to‐noise ratio compared to most other GPCRs that bind diffusible ligands. Additionally, specific amino acid residues in the retinal binding pocket help maintain the extremely low dark noise rate of rhodopsin [97]. Conversely, it has been suggested that dark noise arises from the thermal isomerization of the chromophore in rhodopsin following the deprotonation of the Schiff base linkage, which reduces the energy barrier for thermal isomerization [96]. Thus, dark noise may occur not randomly in rhodopsin but in deprotonated rhodopsin. This hypothesis is supported by observations of the circadian rhythm of dark noise in horseshoe crab photoreceptors, where a reduction in dark noise at night is accompanied by a decrease in external pH in the membrane near rhodopsin [96]. Both current injection into the optic nerves and low pH saline injection into the photoreceptors have been shown to reduce dark noise, reinforcing this hypothesis. Therefore, it can be speculated that local pH environments around rhodopsins in thermosensory neurons could be altered by temperature, enabling rhodopsin activation [62, 96]. Indeed, *Drosophila* larvae and mammalian sperm opsins require 11‐*cis* retinal and tri‐cis retinal isomers, respectively, for thermotaxis [68, 71, 77, 98]. The involvement of multiple opsins and their specific retinal isomers, along with their respective signaling pathways, might be important for their exceptional temperature‐sensing capability [98, 99].

The lipid‐dependent thermal stability of the human β_2_‐adrenergic receptor (β_2_AR) has been reported [100]. Cholesterol, an essential lipid in cell membranes, contributes to membrane stabilization and directly binds to β_2_AR, promoting their functional thermal stabilization. Without the cholesterol analog cholesteryl hemisuccinate (CHS), β_2_AR destabilization occurs upon ligand binding as the temperature increases from 25°C to 42°C. Conversely, with CHS, β_2_AR stability increases with rising temperatures, peaking at 37°C [100]. These findings indicate that lipids enhance the thermal stability of membrane proteins, such as GPCRs, by changing membrane flexibility or interaction with proteins [101, 102, 103], which may significantly impact their thermal sensitivity. For example, cholesterol, anionic lipids such as PIP_2_, sphingolipids, and unsaturated fatty acids and their metabolites can activate, stabilize, or modulate ion channels, including thermo‐TRPs and various types of GPCRs [104, 105, 106]. Recent studies have shown that in *Drosophila* neurons, ether phospholipids in the cell membrane alter the thermal threshold of TRPA1, likely due to changes in the physicochemical properties of the membrane [107]. Additionally, 2‐OH sphingolipids regulate membrane rigidity, which is crucial for the cool temperature sensitivity of ionotropic receptors in *Drosophila* sensory neurons [108]. Thus, it can be speculated that lipids are also important for the thermal stability and responsiveness of GPCRs.

In general, GPCRs exhibit lower thermal stability than rhodopsins and other proteins, making structural analysis challenging [109]. However, the flexible nature of GPCRs may make them sensitive to temperature changes, suggesting their potential function as thermosensors. Various factors contribute to GPCR activation, including conformational changes induced by ligand binding and interactions with intracellular molecules such as G proteins and arrestin, GPCR multimerization, and amino acid phosphorylation [56, 109]. In other words, molecules that stabilize in an active state or reduce the energy barrier for activation can alter the thermal sensitivity of GPCRs. The presence of these factors may be crucial for the thermal activation of GPCRs.

### 1.7 GPCR Signaling Molecules in Temperature Sensation

The thermal sensitivity or dependence of signaling molecules may also play crucial roles in cellular thermosensory processes. In rod cell phototransduction, “discrete noise” arises from amplitude fluctuations of rhodopsin, while “continuous noise” consists of low‐amplitude fluctuations. It is reported that some of this continuous noise is attributed to the spontaneous activation of phosphodiesterase, which operates downstream of rhodopsin [110]. In *Drosophila*, the phototransduction cascade involves the activation of rhodopsins, Gα_q_, PLC (*norpA*), DAG lipase (*inaE*), and TRP and TRPL channels (Figure 4) [53]. The signal transduction pathway is significantly affected by temperature changes. For instance, increasing the temperature over a range from 25°C to 37°C correlates with enhanced PLC activity and membrane dynamics [111], thereby accelerating the kinetics of light activation of photoreceptors [112]. The thermal sensitivity of these signaling molecules is likely linked to the complexity of the rhodopsin‐mediated thermosensation observed in *Drosophila* and mammalian sperm, as well as SRH‐40‐mediated thermosensation in *C. elegans* (Figures 2A,B, and 3C). The activation of PLC and production of DAG and IP_3_ lead to an increase of intracellular calcium that is necessary for regulation of various signaling molecules, including PLC [113, 114] and store‐operated calcium entry [115]. Thus, the involvement of GPCR‐driven intracellular signaling molecules and their potential thermal sensitivity also need to be investigated.

Temperature changes influence several biological responses mediated by β_2_AR signaling, although β_2_AR itself and the binding affinity of norepinephrine do not appear to be thermosensitive. In guinea‐pig atria, the β_2_AR‐dependent function of myofilaments was altered by temperature changes ranging from 24°C to 42°C [116, 117]. Additionally, CREB‐dependent transcription in murine hypothalamic cells through β_2_AR signaling showed significant changes within the physiological fluctuations of core body temperature between 36°C and 38°C [118]. These temperature dependencies in β_2_AR signaling may be attributed to the thermal sensitivity of AC and phosphodiesterase, which synthesize and hydrolyze cAMP, respectively [119, 120].

These examples highlight the complexity of cellular thermosensory mechanisms. Each signal component in the thermosensory pathway may exhibit unique thermal sensitivities and responses, leading to unpredictable overall cellular reactions. Furthermore, the lipid bilayer, where much of signal transduction occurs, undergoes dynamic structural and fluidity changes with temperature fluctuations [121]. Therefore, a comprehensive approach that considers all potential players in signaling pathways is essential for investigating cellular temperature responses. The dual thermosensory mechanism in our model may offer insights into the complex and sophisticated thermosensation observed in animals.

## 2 Conclusions

The role of GPCRs in thermosensation is more complex than that of ion channels, as GPCRs drive intracellular signaling that leads to various cellular outputs. Our dual thermosensory model in *C. elegans* and other animals demonstrated the interaction between GPCRs and thermo‐TRPs within the same cells, indicating that GPCR thermosensors may regulate thermo‐TRPs in many instances. This interaction could represent key features of thermosensation, as it amplifies small signal inputs, resulting in significantly high sensitivity to temperature changes, exemplified by mammalian sperm, which can detect changes as low as 0.00006°C along their body length [76], and *Drosophila* larvae, which can sense changes around 0.005°C/s [122]. Moreover, multiple signaling pathways driven by GPCR activation may influence various cellular functions, beyond the temporary excitation induced by thermosensitive ion channels, providing advantages for chronic responses such as temperature acclimatization and cold tolerance [81]. Therefore, dual thermosensory mechanisms involving both GPCRs and TRP channels may be conserved across various sensory systems in various species.

Currently, there is no molecular evidence that GPCRs are directly activated by temperature changes. Although the flexible structure of GPCRs may allow them to respond to temperature, they cannot be classified as thermosensors unless their direct activation by temperature changes is demonstrated. Moreover, their thermal activation should correlate with temperature‐related physiological responses. Since the stability of GPCRs is regulated by interactions with other components, such as lipids and pH [104], these factors must also be considered. Lipids predominantly modulate membrane proteins, whereas pH change affects all surrounding proteins. It is probable that thermosensitive GPCRs trigger cellular thermosensation through the combined local and global effects of these elements. Elucidating the underlying mechanisms of GPCR‐mediated thermosensation requires comprehensive analyses of receptors and cellular components, the integration of various scientific disciplines and perspectives, and innovative analytical approaches.

## Author Contributions

Kohei Ohnishi and Takaaki Sokabe contributed to the writing of manuscript.

## Conflicts of Interest

The authors declare no conflicts of interest.

## Data Availability

Data sharing is not applicable to this article, as no new data were created or analyzed in this study.
